# Evolutionary patterns of Toll-like receptor signaling pathway genes in the Suidae

**DOI:** 10.1186/s12862-016-0602-7

**Published:** 2016-02-09

**Authors:** Kwame A. Darfour-Oduro, Hendrik-Jan Megens, Alfred L. Roca, Martien A. M. Groenen, Lawrence B. Schook

**Affiliations:** Department of Animal Sciences, University of Illinois, at Urbana-Champaign, Urbana, Illinois 61801 USA; Animal Breeding and Genomics Centre, Wageningen University, Droevendaalsesteeg 1, Wageningen, 6708 PB The Netherlands; University of Illinois Cancer Center, Chicago, Illinois 60612 USA

**Keywords:** Suidae, Selective constraints, Evolution, Signaling, Upstream and downstream genes

## Abstract

**Background:**

The Toll-like receptor (TLR) signaling pathway constitutes an essential component of the innate immune system. Highly conserved proteins, indicative of their critical roles in host survival, characterize this pathway. Selective constraints could vary depending on the gene’s position within the pathway as TLR signaling is a sequential process and that genes downstream of the TLRs may be more selectively constrained to ensure efficient immune responses given the important role of downstream genes in the signaling process. Thus, we investigated whether gene position influenced protein evolution in the TLR signaling pathway of the Suidae. The members of the Suidae examined included the European *Sus scrofa* (wild boar), Asian *Sus scrofa* (wild boar), *Sus verrucosus*, *Sus celebensis*, *Sus scebifrons, Sus barbatus, Babyrousa babyrussa, Potamochoerus larvatus, Potamochoerus porcus and Phacochoerus africanus*.

**Results:**

A total of 33 TLR signaling pathway genes in the Suidae were retrieved from resequencing data. The evolutionary parameter ω (dn/ds) had an overall mean of 0.1668 across genes, indicating high functional conservation within the TLR signaling pathway. A significant relationship was inferred for the network parameters gene position, number of protein-protein interactions, protein length and the evolutionary parameter dn (nonsynonymous substitutions) such that downstream genes had lower nonsynonymous substitution rates, more interactors and shorter protein length than upstream genes. Gene position was significantly correlated with the number of protein-protein interactions and protein length. Thus, the polarity in the selective constraint along the TLR signaling pathway was due to the number of molecules a protein interacted with and the protein’s length.

**Conclusion:**

Results indicate that the level of selective constraints on genes within the TLR signaling pathway of the Suidae is dependent on the gene’s position and network parameters. In particular, downstream genes evolve more slowly as a result of being highly connected and having shorter protein lengths. These findings highlight the critical role of gene network parameters in gene evolution.

**Electronic supplementary material:**

The online version of this article (doi:10.1186/s12862-016-0602-7) contains supplementary material, which is available to authorized users.

## Background

Proteins carry out their biological function within intricate networks of interacting molecules. High throughput techniques such as whole genome sequencing have led to accurate representation of gene networks [1]. Key insights regarding the influence of natural selection on genes can be obtained by taking into account the network topology [2].

The evolutionary rate of a protein (ω), represented by the ratio of the rate of its nonsynonymous substitutions (dn) to the rate of its synonymous substitutions (ds), is used as an indicator of the selective constraints acting on proteins. Studies have indicated that many factors affect the evolutionary rate of genes within gene pathways and networks. For example, gene position [3, 4], protein length [5], the number of protein-protein interactions [6, 7] and codon bias [8, 9] have influence on gene evolution. Signaling pathways mediate the sensing and processing of both extracellular and intracellular stimuli. They rely on receptors that recognize a signaling molecule and trigger a series of events leading to the transmission of signals to the downstream region of the pathway. Within the *Drosophila* Toll and Imd signaling pathways, downstream genes were more conserved, indicating a relatively stronger evolutionary constraints than upstream genes [10]. This observation is consistent with trends reported for the yeast HOG-signaling pathway [11] and the *Caenorhabditis elegans* and *Drosophila* insulin/TOR pathway [12, 13]. In contrast, evolutionary patterns of the Insulin/FOXO signaling pathway across metazoan species genomes indicated that components within the middle of the pathway were rather under stronger purifying selection [9]. Moreover, within the *Drosophila* Ras signaling pathway, upstream genes have lower evolutionary rate, suggesting more constraints, than downstream genes [14]. Obviously, different patterns of selective constraints exist for different signaling pathways. This is expected, as signaling inputs and network architecture vary among the pathways [15].

The TLR signaling pathway represents the best characterized component of the innate immune system [16]. TLR signaling involves the dimerization of receptors in response to pathogenic microbial products. This is followed by the recruitment of various adaptor molecules. The recruitment of these adaptor molecules leads to the recruitment of downstream signaling molecules that activate transcription factors leading to the release of inflammatory cytokines and type I interferons. The TLR signaling is therefore a sequential process. Thus, the effects of pathway parameters such as gene position on the evolutionary rate of genes warrant further analyses.

The TLR signaling pathway has been selectively constrained over time, indicating its essential role for host survival [17]. In humans, population genetic studies have indicated that purifying selection is stronger within TLR adaptors relative to receptors [17]. A recent study on the TLR signaling pathway-related genes from eight vertebrate genomes showed that the selective constraints of genes was negatively correlated with gene position along the TLR signaling pathway [4]. Thus, different components of the TLR signaling pathway appear to differ in their evolutionary rates. Moreover, taxon specific differences among pathway components have been demonstrated for the TLR pathway [16]. New insights relating to the influence of network parameters on the evolutionary rate of genes within the TLR signaling pathway can be gained through studies on closely related species not yet investigated in detail.

Members of the family Suidae have successfully inhabited broad geographic locations including Eurasia, mainland Southeast Asia, island Southeast Asia [18] and the African continent [19]. Given the numerous pathogenic challenges across these diverse environments, the TLR signaling pathway may have been critical in the survival of suids. Suid species are increasingly becoming important biomedical models given their anatomical, physiological and immunogenetics similarities with humans. Comparison of protein sequences of the domestic pig, a member of the Suidae, to human orthologues revealed 112 positions where the domestic pig protein has the same amino acid that is implicated in a human disease [20]. Thus, findings regarding suid TLR signaling genes evolutionary rates can be relevant to their hominid orthologues. The availability of a complete porcine genome and resequencing libraries of species within the family Suidae provide an opportunity to investigate the evolution of the TLR signaling pathway genes within the context of gene position and other network parameters. The suid species have evolved over a relative short time span of 1–10 million years [21]. The low divergence among these species indicate that synonymous substitutions are not likely to be saturated, making the estimation and comparison of selective pressure (dn/ds) among genes less prone to bias. This low divergence among the species also ensures reliability in aligning TLR sequences for subsequent analysis.

In the current study, our goal was to investigate the evolutionary constraints of genes within the TLR signaling pathway of the family Suidae. We sought to investigate the extent to which patterns of protein evolution observed in other organisms extend to members of the family Suidae. We hypothesized that there is a polarity of purifying selection along the TLR signaling pathway within the family Suidae.

We tested our hypothesis by answering the following questions 1) is there a relationship between the strength of purifying selection and gene position in the TLR signaling pathway and; 2) are there any network parameters that contribute to the polarity in the strength of purifying selection?

## Methods

### Ethics statement

Blood samples were obtained from captive-held suid species by qualified individuals at each institution, according to their respective animal welfare oversight governance and institutional permits (See Additional file 1: Table S1). All animal procedures were conducted in accordance with the guidelines approved by the American Society of Mammalogists [22], and approved by the University of Illinois’ Institutional Animal Care and Use Committee (IACUC; Protocol numbers 99252 and 04006). Sources of animals are provided in Additional file 1: Table S1.

### DNA extraction and sequencing

DNA was extracted from whole blood using the QIAamp DNA blood spin kit (Qiagen Sciences) and quantity and quality parameters were performed using the Qubit 2.0 fluorometer (Invitrogen) and visualized using a 1 % agarose gel. Library construction and re-sequencing of individual members of the family Suidae were done with 1–3 μg of genomic DNA according to the Illumina library prepping protocols. The library insert size was 300–500 bp and sequencing was performed using a 100 paired-end sequencing kit. All DNA samples were sequenced to approximately 8x depth. Quality trimmed reads (phred quality > 20, minimum length of pairs of reads = 40 bp) were aligned to the *Sus scrofa* reference genome build 10.2 using the unique alignment option of Mosaik Aligner (V.1.1.0017). The aligned reads from each of the animals (resequencing libraries) together with the *Sus scrofa* reference genome were stored as bam files for each individual animal.

### Ortholog identification

**Fig. 1:**
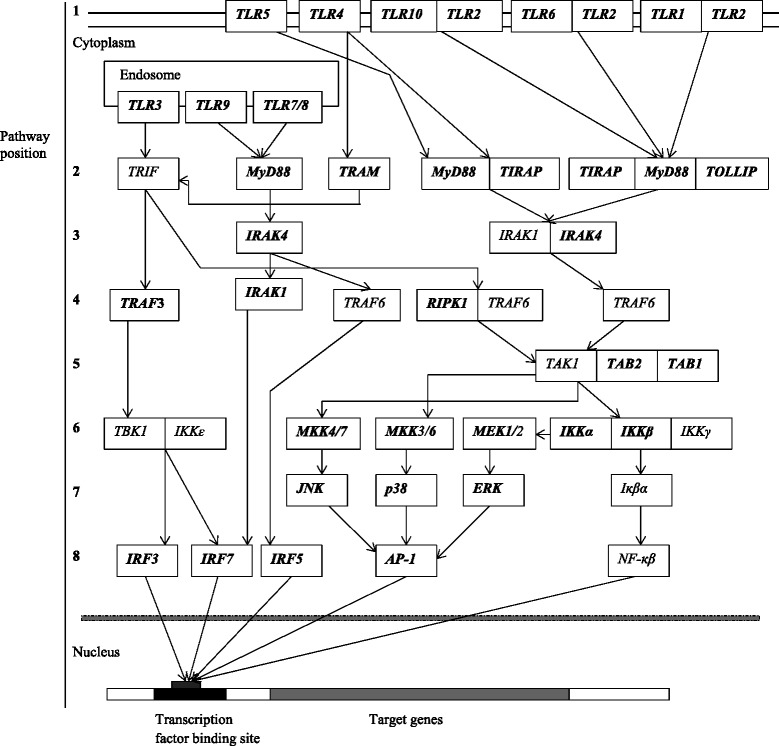
The TLR signaling pathway genes. Redrawn following [4], with modification to include *TLR10*. The direction of signal transduction is indicated by the arrows. TLRs 3, 7, 8, and 9 are included in the first pathway position category. The numbers on the left side represent the position of the pathway genes. Genes used in this study are shown in bold

Genes involved in the TLR signaling pathway (KEGG database pathway: ssc04620) were examined for this study (Fig. 1). In addition *TLR10,* which is not found in the KEGG database TLR signaling pathway, was included since it cooperates with *TLR2*, senses lipopeptides [23] and activates the TLR signaling pathway through association with *MyD88* [24]. The genomic coordinates of *Sus scrofa* genes within the TLR signaling pathway (Additional file 2: Table S2) were obtained from the Ensembl database (http://www.ensembl.org). Based on these genomic coordinates, sequences of gene orthologs were then retrieved from aligned bam files (Illumina resequencing data for suid species aligned against *Sus scrofa* genome assembly 10.2) for *Sus scrofa* (*Sus scrofa* Europe and *Sus scrofa* Asia), *Sus verrucosus*, *Sus celebensis*, *Sus scebifrons*, *Sus barbatus*, *Babyrousa babyrussa*, *Potamochoerus larvatus*, *Potamochoerus porcus* and *Phacochoerus africanus* to identify gene orthologs. The resulting sequences for each species were then screened against the genome of *Sus scrofa* using blast to ensure similarity with the *Sus scrofa* genes. To obtain the coding sequence of genes for each species, exonic regions were retrieved based on genomic coordinates of exons from the *Sus scrofa* gene transcripts (see Additional file 2: Table S2 for accession numbers of mRNA sequences/gene transcripts) and concatenated. If a gene was found to have more than one transcript, the longest transcript was chosen for analysis. Coding sequences were aligned using ClustalW 1.81 [25]. Aligned sequences are provided in Additional file 3.

Some of the coding sequences had missing nucleotides. The following criteria were used to discard such sequences: (1) sequences with more than 50 % of missing amino acid residues; and (2) aligned sequences absent in any one of the species involved in this study. Consequently, the final data set was composed of 33 genes (Additional file 4: Table S3).

### Impact of natural selection

**Fig. 2:**
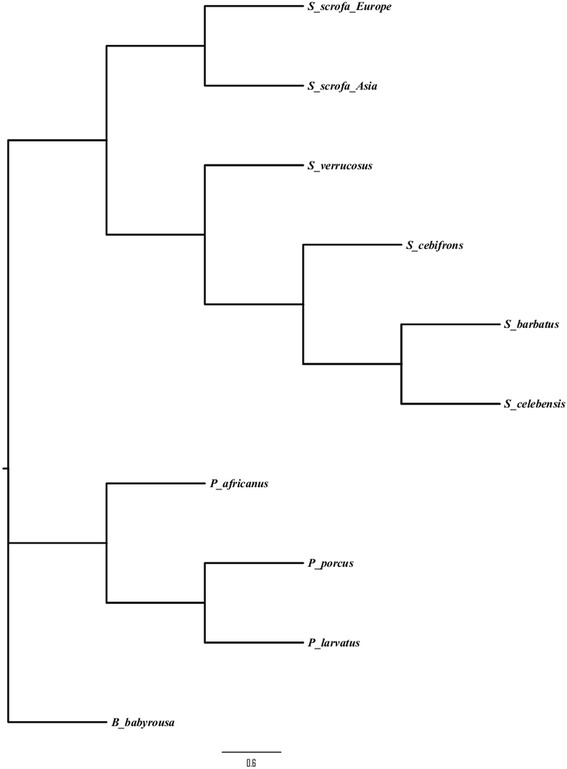
Phylogenetic relationships among species of the family Suidae obtained from near complete genome data of each species. The posterior probability at each node is 1

The impact of natural selection on genes within the TLR signaling pathway was determined by estimating nonsynonymous substitution rate (dn), synonymous substitution rate (ds) and their ratio (ω = dn/ds) using the M0 model implemented in the program CODEML from the PAML package version 4 [26, 27]. To determine whether specific codon positions were under positive selection within gene orthologs, CODEML site model M1a, a nearly neutral evolution model where sites are assumed to be evolving under either purifying selection (ω < 1) or neutral evolution (ω = 1) was compared to model M2a that allows positive selection (ω >1) among sites. M7, which allows sites to evolve under either purifying selection or neutrally, was compared to model M8, which allows for positively selected sites (ω >1) . Models were compared using a likelihood ratio test (LRT). The F3x4 model of codon frequencies was used for the analyses. Models were run in duplicates with ω of 0.5 and 1.5 to increase the probability of convergence of model parameters. Multiple testing for positive selection on genes was corrected for by conducting a false discovery rate (FDR) test [28] at a q value of 0.05. The phylogenetic relationship among the species of Suidae (Fig. 2) inferred from near complete genome sequences of each species [18, 21] was used for the analysis involving the CODEML program.

### Network level analysis

To determine whether the evolution of molecules within each gene was affected by network structure, network parameters were computed for each gene and were correlated with each evolutionary parameters estimated by model M0. The number of protein-protein interactions (PPI) for proteins encoded by each gene was determined from the *Sus scrofa* interaction network in the STRING database (http://string.embl.de/). STRING is a database of known and predicted interactions which include direct (physical) and indirect (functional) associations from various sources including high throughput experiments and genomic contexts [29]. The magnitude for codon bias for individual genes was measured by the Effective Number of Codons (ENC). The codon usage bias of each orthologous gene group was measured as the mean of ENC of each species gene. ENC values of each gene were obtained using DNASP software [30]. Bivariate correlation analysis between network parameters (pathway position within network, protein length, codon bias, connectivity (number of protein-protein interactions (PPI)) and length of 3′UTR (L3′UTR)) and evolutionary parameters (ω, dn, ds) were conducted using Spearman’s rank correlations. A false discovery rate (FDR) test [28] was performed to correct for multiple testing for correlations controlling for q value at 0.05. Bivariate correlations and corrections for multiple testing were performed using SAS software (SAS/STAT, version 9.1.3, SAS Institute Inc., Cary, NC). Figures illustrating bivariate correlations were obtained with the package R (http://www.rproject.org).

### Multivariate analyses

The two multivariate analyses methods (partial correlation and path analysis) were used to determine whether the observed bivariate correlations were due to direct or indirect influences. Partial correlation analysis measures the strength of relationship between two variables, while holding one or more variables constant. Path analysis estimates direct and indirect relationships under a user defined causal model. For the path analysis, pathway position, PPI, protein length, ENC and L3′UTR were considered as exogenous variables whereas ω and dn were considered as endogenous variables. Prior to performing the path analysis, data were log-transformed to improve normality. All statistics related to path analysis were calculated by the lavaan package in R.

## Results

Orthologs of 33 *Sus scrofa* TLR signaling pathway genes were identified for 10 members of the family Suidae. A total of 330 sequences of the TLR signaling pathway genes ranging from 351 to 3153 nucleotides were therefore used in analysis. The orthologous sequences were aligned for a series of analyses including test for selection pressures acting on genes, bivariate correlations and multivariate analysis.

### Analysis of protein sequence evolution

In order to estimate the selective pressure acting on genes within the suid TLR signaling pathway, the M0 model, which provides a single estimate of ω across all codons and lineages, was utilized. The ω values ranged from 0.0001 (*MKK6, MEK1, MAPK1, MAPK9 and MAPK14*) to 1.0544 (*TLR10*) (Additional file 4: Table S3). The mean ω was 0.1668. To test for positive selection, the models M1a (nearly neutral) vs M2a (positive selection); M7 (nearly neutral) vs M8 (positive selection) were used. The two tests were in agreement for all genes (Additional file 5: Table S4) and demonstrated that a proportion of sites within *TLR1, TLR2, TLR6 and IRAK4* (Additional file 5: Table S4) were under positive selection. However, *TLR1* was the only gene that had sites under positive selection after correcting for multiple testing at q = 0.05. Thus, results here indicated that genes within the TLR signaling pathway have evolved under strong functional constraint.

### Relationship between evolutionary rates and pathway position

**Fig. 3:**
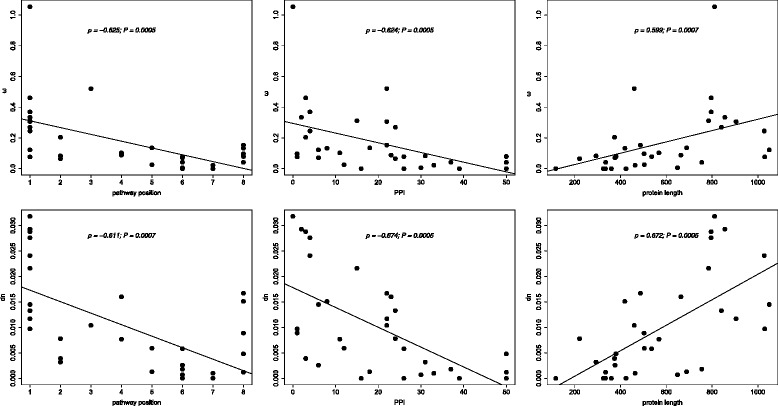
ω and dn versus pathway position, number of protein-protein interactions (PP1) and protein length. All relationships are significant. Continuous lines represent regression lines

The relationship between the evolutionary parameter ω of genes and their position within the TLR signaling pathway was determined using a Spearman’s rank correlation test between the two variables. The ω values were negatively correlated with pathway position (Spearman’s rank correlation coefficient *ρ* = −0.6250; *P* = 0.0005 after FDR correction; Additional file 6: Table S5 and Fig. 3) indicating that downstream genes are under stronger purifying selection than upstream genes. The robustness of the correlation between ω and pathway position was tested by removing the highest ω value (ω = 1.05445 for *TLR10*) and re-estimating the correlation. This was done to ensure that results were not influenced by high ω values. The correlation remained significant (*ρ* = −0.5985; *P* = 0.0017 after FDR correction). To test for the evolutionary parameter accounting for the ω differences among genes, the correlation between pathway position and dn, pathway position and ds was also determined. The dn was negatively correlated with the pathway position (Spearman’s rank correlation coefficient *ρ* = −0.6110; *P* = 0.0007 after FDR correction; Additional file 6: Table S5 and Fig. 3). There was no significant correlation between ds and pathway position (Spearman’s rank correlation coefficient *ρ* = −0.0990; *P* = 0.7117). These results indicated that the decrease in ω (increase in the strength of purifying selection) along the TLR signaling pathway (from upstream with TLRs being the most upstream genes to downstream of the TLR genes) is attributable to a decrease in dn (the rate of nonsynonymous substitution).

### Relationship between evolutionary rates and network parameters

Network parameters other than pathway position may influence evolutionary parameters (ω, dn, ds) and account for inferred polarity in ω and dn. In order to determine such relationships, Spearman’s rank correlation test was performed between PPI of proteins (determined from the STRING database with settings *Sus scrofa*, highest confidence of 0.700 and no more than 50 interactors) encoded by genes within the TLR signaling pathway, protein length, codon bias measured as effective number of codons (ENC), L3′UTR region and the evolutionary parameters (Additional file 6: Table S5 and Fig. 3). The ω values were negatively correlated with PPI (Spearman’s rank correlation coefficient *ρ* = −0.6240; *P* = 0.0005 after FDR correction) and positively correlated with protein length (Spearman’s rank correlation coefficient *ρ* = 0.5990; *P* = 0.0007 after FDR correction). Similarly, dn values were negatively correlated with PPI (Spearman’s rank correlation coefficient *ρ* = −0.6740; *P* = 0.0005 after FDR correction) and positively correlated with protein length (Spearman’s rank correlation coefficient *ρ* = 0.672; *p* = 0.0005 after FDR correction) indicating that apart from pathway position, the number of protein-protein interactions a given protein is involved in and its protein length influence it evolution. In addition, ds was also negatively correlated with ENC (Spearman’s rank correlation coefficient *ρ* = −0.4650; *p* = 0.0163 after FDR correction) indicating stronger selection based on codon usage in genes with high codon bias than genes with low codon bias [31].

Since pathway position, PPI and protein length are intercorrelated and are each correlated with evolutionary parameters (ω and dn) (Additional file 6: Table S5), observed associations of these network parameters with the evolutionary parameters could be indirect (correlation between 2 parameters due to their both being correlated with a third parameter). Thus, to distinguish between direct and indirect effects, multivariate analysis (partial correlation and path analysis) were utilized. Partial correlation analysis revealed that when controlling for PPI, the correlation between ω and pathway position remained significant (*ρ* = −0.431, *P* = 0.014). When controlling for protein length, the correlation between ω and pathway position remained significant (*ρ* = −0.386, *P* = 0.029). Thus, gene position within the TLR signaling pathway directly influenced the evolution of genes in the TLR signaling pathway. The correlation between ω and PPI while controlling for pathway position remained significant (*ρ* = −0.429, *P* = 0.014). It also remained significant (*ρ* = −0.452, *P* = 0.009) when controlling for protein length. This result indicated that PPI is an important factor affecting the evolution of genes within the TLR signaling pathway. The correlation between ω and protein length was not significant (*ρ* = 0.323, *P* = 0.072) when pathway position was held constant indicating that the correlation between ω and protein length was rather mediated by pathway position. Nonsynonymous (dn) substitution rates were not significantly (*ρ* = −0.306, *P* = 0.089) correlated with pathway position when controlling for protein length. In contrast, the correlation between dn and PPI remained significant (*ρ* = −0.506, *P* = 0.003) when controlling for protein length. If pathway position was held constant, the correlation between dn and PPI remained significant (*ρ* = −0.600, *P* = 0.003). Correlation between dn and protein length was still significant (*ρ* = 0.456, *P* = 0.009) after controlling for pathway position and remained significant (*ρ* = 0.504, *P* = 0.003) if controlling for PPI. Results for the relationship between dn values and network parameters indicated that the number of protein-protein interactions and protein length have direct effects on the rate of dn evolution.

**Fig. 4:**
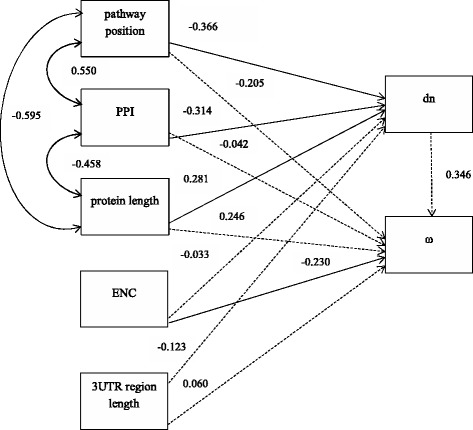
The causal model used to analyze the relationship among evolutionary and network parameters. The network parameter pathway position, number of protein-protein interactions (PPI), protein length, codon bias (measured as effective number of codons (ENC)) and length of the 3′UTR region are considered as exogenous variables. Continuous and dashed lines represent significant and nonsignificant relationships, respectively. Single-headed arrows indicate causal relationship between variables. Double-headed arrows indicate correlations between exogenous variables. Numbers on the arrows represent the standardized regression weights

The relationship between evolutionary parameters (ω and dn) and the network parameters (pathway position, PPI, protein length, ENC and L3′UTR) were further analyzed using path analysis. As depicted in Fig. 4, path analysis indicated that dn values were affected by gene position within the TLR signaling pathway (standardized path coefficient, β = −0.366, *P* = 0.008). Pathway position had the largest direct effect on dn. The dn values were negatively associated with the number of protein-protein interactions (β = −0.314, *P* = 0.011) and positively associated with protein length (β = 0.281, *P* = 0.029). ω values were only associated with ENC (β = −0.230, *P* = 0.044). Thus pathway position, the number of protein interactions and protein length were factors that influenced TLR signaling pathway dn substitution rates in the Suidae.

## Discussion

Our results support the hypothesis that there is polarity of purifying selection along the TLR signaling pathway (from TLRs as upstream genes to genes downstream of TLRs) within the family Suidae with purifying selective pressure increasing along the pathway. Protein-protein interactions and protein length accounted for this polarity in the strength of purifying selection, where number of interactors of TLR signaling protein molecules increases and protein length decreasing along the TLR signaling pathway. The evolutionary parameter influenced by these network parameters was dn (nonsynonymous substitution rate), which is actually the metric of selective pressure [10]. Fewer amino acid substitutions were tolerated along the signaling pathway reflective of stronger purifying selection. In a previous study on the evolution of TLR signaling pathway-related genes from eight vertebrate genomes (human, chicken, western clawed frog, chimpanzee, macaque, mouse, cow and zebrafish) [4], selective constraint was negatively correlated with gene position in the signaling pathway and dn was also influenced by position and protein length in a similar manner as in the present study. These similarities indicate that some patterns revealed across the eight vertebrate genomes are also seen in the closely related suid species involved in this study.

Several other studies conducted on various biological pathways have revealed a polarity in the strength of purifying selection. Upstream genes revealed higher constrains than downstream genes in the carotenoid biosynthesis pathway [32, 33], in the plant terpenoid biosynthesis pathway [1] and in the dopamine catabolic pathway across mammals [34]. Furthermore, in the *Drosophila* Ras signaling pathway, upstream genes evolve under higher levels of selective constrains relative to their downstream counterparts [14] The relatively slow rate of evolution of upstream genes reflects these genes being required for a wider range of end products and therefore being more pleiotropic [35, 36] and in the case of biosynthesis pathways, upstream genes exert a higher control over the flux of metabolites throughout the pathway [37]. A negative correlation between strength of purifying selection and gene position within pathway, as inferred in this study, have also been reported for the *Drosophila* Toll and Imd signaling pathways [10], *Caenorhabditis* insulin/TOR signal transduction pathway [12] and the yeast HOG pathway [11]. This pattern of selective constraint might be attributed to purifying selection acting to maintain the function of downstream signal transduction elements and a concentration of adaptive changes in the upstream genes due to their interaction with external environment [12]. In this study, *TLR1* was the only gene inferred to be under positive selection after FDR correction. Therefore, adaptive changes were not prominent within upstream genes of the TLR signaling pathway to result in the inferred pattern of selective constraints.

With the inferred significantly negative correlation between pathway position and the evolutionary parameters ω and dn, a negative correlation between pathway position and number of protein-protein interactions for a protein, as indicated in this study, implies a role for the number of protein-protein interactions in the polarity of selective constraints. This negative relationship is indicative of increasing selective constraints for proteins along the TLR signaling pathway, as a result of increase in the number of interactors along the signaling pathway. Evidence for higher connectivity (protein-protein interactions) of downstream genes has also been demonstrated for the human signal transduction network [2]. Within metabolic pathways, connectivity plays a major role in constraining evolutionary rates with proteins interacting with more proteins being subject to stronger selective constraints [6, 38, 39]. The increasing number of interactors along the TLR signaling pathway indicates the essential role of protein molecules downstream of TLRs in propagating and amplifying signals initiated by pathogen binding of TLRs and the role of downstream protein molecules in other functions apart from TLR signaling. This places stronger selective constraint on the intracellular signaling protein molecules. The finding of an effect of connectivity on dn in this study is in contrast to the study of Song et al. [4] that did not show a significant influence of connectivity on dn in a multivariate analysis of factors influencing evolution of genes in the TLR signaling pathway across 8 vertebrate genomes. A possible explanation for this difference may be that the use of suid species that are more closely related in this study than species involved in the study of Song et al. [4] led to greater precision in the estimation of dn and thus the relationship between connectivity and dn was more evident.

The finding of a significant negative correlation between protein length and pathway position in this study corroborates studies in the insulin/TOR signaling of *Drosophila* [3] and indicated that downstream genes encode shorter and more actively translated proteins [3]. Given that partial correlation and pathway analysis were both in agreement as to a positive relationship between protein length and dn, protein length appears to be a factor responsible for the polarity in dn along TLR signaling pathway. Thus, along the TLR signaling pathway, the decrease in protein length is associated with decreases in amino acid substitutions. A similar pattern has been reported for the woody perennial plant *Populus tremula*, where protein length is the main factor affecting selective constraints, with purifying selection weaker in genes with longer coding regions [40]. A possible explanation for the relationship between purifying selection and protein length is that selection at more than one site should cause an overall reduction in the effectiveness of selection (Hill-Robertson effect) [41, 42]. In that case, for longer proteins, which may have many sites under selection simultaneously [40], there will be a reduced efficiency in natural selection.

Though path analysis indicated a negative association between ω and ENC, pathway position and ENC were not significantly correlated, therefore ENC cannot explain correlation between selective constraints and ENC. In this study, length of the gene 3UTR region did not have a relationship with any evolutionary parameter in disagreement with studies in the Toll/imd pathway in the Drosophila Toll and Imd signaling pathways [10] and human and mouse miRNA target prediction data [43].

Genes within the signaling pathway had an average ω value < 1, indicating that the TLR signaling pathway is selectively constrained across members of the Suidae. This is in keeping with the essential role of the pathway in innate immunity and host survival. Selectively constrained regions within the genome are likely to be functionally important [44]. The strongest selective constraints inferred for the MAP kinases (*MKK6, MEK1, MAPK1, MAPK9, MAPK14*) with dn substitution rates of zero for these genes suggest their involvement in critical roles during TLR signaling. The MAP kinases cascade components play important role in the production of proinflammatory mediators [45]. Recently, it has been demonstrated that MAP kinases play a role in agonist dependent regulation of cognate TLR mRNA levels [46]. MAP kinases are required for the regulation of cellular development and differentiation processes [47, 48]. The conservation of the MAP kinase genes in this study could therefore be attributed to their involvement in many processes. The stronger purifying selection detected in downstream genes of TLRs relative to upstream genes suggests that there is a greater need to protect the integrity of proteins downstream [17]. Thus, genes downstream might be essential for survival of suid species involved in this study. TLR signaling evolution is partly dictated by the need to conserve the capability to recognize specific pathogen signature [49]. For the homogenous group of suid species involved in this study, the evolutionary dynamics of the TLR signaling pathway as revealed in this study may be reflective of the range of related pathogens that adversely affected them during their evolution. Our study has provided insight into factors that have shaped the present day TLR signaling pathway genes of the suid species, a convenient number of which are still present in the world [50]. As the wild suid species are among animals at the greatest risk of declines due to cross species transmission of disease with their domesticated counterparts (pigs) [51], evolutionary analysis of the TLR signaling pathway as presented here can aid in identifying genes that are essential in mediating the immune response to disease causing bacteria and viruses. For example, a protein that has a higher connectivity and selectively constrained within the TLR signaling pathway would be expected to be of functional relevance in terms of disease prevention.

## Conclusion

The findings here provide evidence of the role of network topology in the polarity of purifying selection along the TLR signaling pathway of the Suidae. Our work indicates that the Suidae are an attractive system of closely related but well characterized species to study the evolutionary dynamics of TLR signaling genes. Furthermore, our work opens other avenues of research including the use of the Suidae in understanding patterns of molecular evolution in other important pathways such as metabolic and gene-regulation pathways.

### Availability of supporting data

All supporting data are included as additional files in the form of Additional files 1, 2, 3, 4, 5 and 6.

## Additional files

Additional file 1: Table S1. Sample information. (DOCX 12 kb) Additional file 2: Table S2. Genes of the *Sus scrofa* TLR signaling pathway used in querying genomes of other species of the Suidae. (DOCX 13 kb) Additional file 3:
**Multiple sequence alignments of the TLR signaling pathway orthologs across the suid species.** Asteriks indicate same nucleotide and “-“ represents missing nucleotide. (TXT 1867 kb) Additional file 4: Table S3. Summary statistics for genes. (DOCX 14 kb) Additional file 5: Table S4. Results of codon based test for positive selection. (DOCX 13 kb) Additional file 6: Table S5. Bivariate correlations among variables. (DOCX 13 kb)
