# Supplementary material for: Evolutionary patterns of Toll-like receptor signaling pathway genes in the Suidae
Source: BMC Evol Biol. 2016 Feb 9;16:33. doi: 10.1186/s12862-016-0602-7 (PMC4748524; doi:10.1186/s12862-016-0602-7)
Supplement: Additional file 1: Table S1. — Sample information. (DOCX 12 kb) [file 12862_2016_602_MOESM1_ESM.docx]

**Table S1:** Sample information

| **Suid species** | **Source** | **DNA samples provider** | **Institution** |
| --- | --- | --- | --- |
| *Potomochoerus larvatus* | San Diego Zoo, USA | Oliver Ryder | San Diego Zoo Institute for Conservation Research, USA |
| *Potomochoerus porcus* | San Diego Zoo, USA | Oliver Ryder |  |
| *Babyrousa babyrussa* | San Diego Zoo, USA | Oliver Ryder |  |
| *Sus cebifrons* | San Diego Zoo, USA | Oliver Ryder |  |
| *Sus celebensis* | Roslin, UK | Alan Archibald | Roslin Institute and the Royal (Dick) School of Veterinary Studies, University of Edinburgh, UK |
| *Sus barbatus* | Sumatra, Indonesia | Gono Semiadi | Puslit Biologi, LIPI, Indonesia |
| *Sus verucosus* | Surabaya Zoo, Indonesia | Gono Semiadi |  |
| *Phacochoerus africanus* | Omaha’s Zoo, USA | Cheryl Morri | Omaha’s Henry Doorly Zoo, USA |
| *Sus scrofa* Europe | Meinweg; Roerdalen, Netherlands | Richard Crooijmans | Animal Breeding and Genetics Group, Wageningen University, The Netherlands |
| *Sus scrofa* Asia | South China, China | Ning Li | China Agricultural University, China |
