# Supplementary material for: Evolutionary patterns of Toll-like receptor signaling pathway genes in the Suidae
Source: BMC Evol Biol. 2016 Feb 9;16:33. doi: 10.1186/s12862-016-0602-7 (PMC4748524; doi:10.1186/s12862-016-0602-7)
Supplement: Additional file 2: Table S2. — Genes of the Sus scrofa TLR signaling pathway used in querying genomes of other species of the Suidae. (DOCX 13 kb) [file 12862_2016_602_MOESM2_ESM.docx]

**Table S2:** Genes of the *Sus scrofa* TLR signaling pathway used in querying genomes of other species of the Suidae

| **Gene** | **Genomic coordinates** | **mRNA accession numbers/Transcript ID** |
| --- | --- | --- |
| *TLR1* | 8: 31,627,788-31,635,628 | NM_001031775 |
| *TLR2* | 8: 79,824,541-79,834,592 | NM_213761 |
| *TLR3* | 15: 53,840,783-53,852,536 | NM_001097444 |
| *TLR4* | 1: 289,775,822-289,785,847 | NM_001113039 |
| *TLR5* | 10: 21,886,309-21,905,776 | NM_001123202 |
| *TLR6* | 8: 31,641,826-31,660,823 | NM_213760 |
| *TLR7* | X: 10,450,659-10,476,655 | NM_001097434 |
| *TLR8* | X: 10,500,862-10,512,940 | NM_214187 |
| *TLR9* | 13: 37,647,229-37,652,020 | NM_213958 |
| *TLR10* | 8: 31,604,732-31,615,025 | NM_001030534 |
| *MyD88* | 13: 25,181,051-25,185,351 | NM_001099923 |
| *TIRAP* | 9: 59,051,622-59,056,203 | ENSSSCT00000028018 |
| *TRAM* | 4: 70,399,155-70,429,614 | ENSSSCT00000006787 |
| *IRAK4* | 5: 77,646,641-77,671,387 | NM_001112693 |
| *TRAF3* | 7: 128,907,875-129,006,770 | ENSSSCT00000002804 |
| *RIPK1* | 7: 1,921,742-1,945,193 | ENSSSCT00000001101 |
| *TAB1* | 5: 6,122,718-6,146,864 | NM_001244067 |
| *TAB2* | 1: 18,852,325-18,903,792 | ENSSSCT00000004545 |
| *IKKα* | 14: 120,756,666-120,854,124 | NM_001114279 |
| *IKKβ* | 17: 13,063,170-13,101,996 | ENSSSCT00000007699 |
| *MKK6* | 12: 11,139,492-11,264,120 | ENSSSCT00000018783 |
| *MKK4* | 2: 59,331,232-59,409,597 | ENSSSCT00000019611 |
| *MKK7* | 2: 71,697,110-71,708,586 | ENSSSCT00000014840 |
| *MEK1* | 1: 182,175,684-182,177,853 | ENSSSCT00000023493 |
| *MAPK1* | 14: 53,590,167-53,614,842 | ENSSSCT00000011042 |
| *MAPK9* | 2: 79,823,591-79,881,875 | ENSSSCT00000035631 |
| *MAPK10* | 8: 141,997,575-142,137,195 | ENSSSCT00000010107 |
| *MAPK14* | 7: 36,725,707-36,795,310 | ENSSSCT00000001734 |
| *IRF3* | 6: 50,430,671-50,436,164 | NM_213770 |
| *IRF5* | 18: 20,747,732-20,760,161 | ENSSSCT00000018043 |
| *IRF7* | 2: 299,444-302,179 | NM_001097428 |
| *FOS* | 7: 104,293,657-104,297,121 | NM_001123113 |
| *JUN* | 6: 141,230,121-141,233,597 | NM_213880 |
