# Supplementary material for: Evolutionary patterns of Toll-like receptor signaling pathway genes in the Suidae
Source: BMC Evol Biol. 2016 Feb 9;16:33. doi: 10.1186/s12862-016-0602-7 (PMC4748524; doi:10.1186/s12862-016-0602-7)
Supplement: Additional file 4: Table S3. — Summary statistics for genes. (DOCX 14 kb) [file 12862_2016_602_MOESM4_ESM.docx]

**Table S3:** Summary statistics for genes

| Gene | **ω** | ***dn*** | ***ds*** | **Position** | **PPI** | **ENC** | **PLENGTH** | **L3’UTR** |
| --- | --- | --- | --- | --- | --- | --- | --- | --- |
| *TLR1* | 0.36932 | 0.0276 | 0.0747 | 1 | 4 | 53.28 | 796 | 181 |
| *TLR2* | 0.31229 | 0.0216 | 0.069 | 1 | 15 | 54.71 | 785 | 192 |
| *TLR3* | 0.30699 | 0.0117 | 0.038 | 1 | 22 | 54.28 | 905 | 218 |
| *TLR4* | 0.26995 | 0.0133 | 0.0493 | 1 | 24 | 53.54 | 841 | 806 |
| *TLR5* | 0.33407 | 0.0293 | 0.0878 | 1 | 2 | 52.27 | 856 | 1516 |
| *TLR6* | 0.46067 | 0.0288 | 0.0624 | 1 | 3 | 53.37 | 796 | 468 |
| *TLR7* | 0.12225 | 0.0145 | 0.1187 | 1 | 6 | 54.58 | 1050 | 655 |
| *TLR8* | 0.24534 | 0.0241 | 0.0984 | 1 | 4 | 56.33 | 1028 | 53 |
| *TLR9* | 0.0764 | 0.0097 | 0.1271 | 1 | 1 | 35.82 | 1030 | 167 |
| *TLR10* | 1.05445 | 0.0318 | 0.0301 | 1 | 0 | 52.49 | 811 | 272 |
| *MyD88* | 0.08294 | 0.0032 | 0.0384 | 2 | 31 | 45 | 293 | 1641 |
| *TIRAP* | 0.0655 | 0.0078 | 0.1197 | 2 | 24 | 41.24 | 221 | 0 |
| *TRAM* | 0.20405 | 0.0039 | 0.0192 | 2 | 3 | 55.13 | 374 | 111 |
| *IRAK4* | 0.52095 | 0.0104 | 0.0199 | 3 | 22 | 50.19 | 460 | 759 |
| *TRAF3* | 0.10269 | 0.0077 | 0.0747 | 4 | 11 | 43.56 | 568 | 4976 |
| *RIPK1* | 0.08849 | 0.016 | 0.181 | 4 | 23 | 39.77 | 664 | 1704 |
| *TAB1* | 0.02593 | 0.0059 | 0.2261 | 5 | 12 | 37.37 | 504 | 895 |
| *TAB2* | 0.1354 | 0.0013 | 0.0098 | 5 | 18 | 52.19 | 689 | 1894 |
| *IKKα* | 0.0417 | 0.0018 | 0.0422 | 6 | 37 | 52.77 | 755 | 548 |
| *IKKβ* | 0.00611 | 0.0007 | 0.1187 | 6 | 30 | 38.85 | 649 | 774 |
| *MKK6* | 0.0001 | 0 | 0.0386 | 6 | 16 | 53.84 | 334 | 277 |
| *MKK4* | 0.07146 | 0.0026 | 0.0369 | 6 | 6 | 52.7 | 376 | 2455 |
| *MKK7* | 0.07876 | 0.0058 | 0.074 | 6 | 26 | 43.74 | 536 | 0 |
| *MEK1* | 0.0001 | 0 | 0.0859 | 6 | 26 | 43 | 117 | 0 |
| *MAPK1* | 0.0001 | 0 | 0.0455 | 7 | 50 | 51.73 | 325 | 634 |
| *MAPK9* | 0.0001 | 0 | 0.0311 | 7 | 39 | 53.73 | 424 | 604 |
| *MAPK10* | 0.02238 | 0.001 | 0.0465 | 7 | 33 | 54.76 | 464 | 3555 |
| *MAPK14* | 0.0001 | 0 | 0.0299 | 7 | 50 | 53.59 | 360 | 2288 |
| *IRF3* | 0.13262 | 0.0151 | 0.114 | 8 | 8 | 40.63 | 419 | 68 |
| *IRF5* | 0.0975 | 0.0089 | 0.0908 | 8 | 1 | 39.74 | 503 | 1138 |
| *IRF7* | 0.15374 | 0.0167 | 0.1086 | 8 | 22 | 36.93 | 487 | 48 |
| *FOS* | 0.07942 | 0.0048 | 0.0608 | 8 | 50 | 43.3 | 380 | 830 |
| *JUN* | 0.04095 | 0.0012 | 0.0305 | 8 | 50 | 37.48 | 335 | 1290 |
