# Supplementary material for: Evolutionary patterns of Toll-like receptor signaling pathway genes in the Suidae
Source: BMC Evol Biol. 2016 Feb 9;16:33. doi: 10.1186/s12862-016-0602-7 (PMC4748524; doi:10.1186/s12862-016-0602-7)
Supplement: Additional file 5: Table S4. — Results of codon based test for positive selection. (DOCX 13 kb) [file 12862_2016_602_MOESM5_ESM.docx]

**Table S4.** Results of codon based test for positive selection

| Gene | LnM1a | LnM2a | LnM7 | LnM8 | 2(LnM2a-LnM1a) | 2(LnM8-LnM7) |
| --- | --- | --- | --- | --- | --- | --- |
| *TLR1* | -3884.4 | -3872.6 | -3884.6 | -3872.6 | 23.6*† | 24*† |
| *TLR2* | -3758.0 | -3753.6 | -3758.2 | -3753.6 | 8.8* | 9.2* |
| *TLR3* | -4016.4 | -4016.4 | -4016.4 | -4016.4 | 0 | 0 |
| *TLR4* | -3832.2 | -3832.0 | -3832.3 | -3832.0 | 0.4 | 0.6 |
| *TLR5* | -4259.9 | -4259.5 | -4259.9 | -4259.5 | 0.8 | 0.8 |
| *TLR6* | -3856.1 | -3852.0 | -3856.4 | -3852.0 | 8.2* | 8.8* |
| *TLR7* | -5104.2 | -5104.2 | -5104.2 | -5104.2 | 0 | 0 |
| *TLR8* | -5085.7 | -5084.2 | -5085.8 | -5084.2 | 3 | 3.2 |
| *TLR9* | -4586.9 | -4586.9 | -4586.9 | -4586.9 | 0 | 0 |
| *TLR10* | -3828.6 | -3828.2 | -3828.6 | -3828.2 | 0.8 | 0.8 |
| *MyD88* | -1238.3 | -1238.3 | -1238.3 | -1238.3 | 0 | 0 |
| *TIRAP* | -1012.8 | -1012.8 | -1012.8 | -1012.8 | 0 | 0 |
| *TRAM* | -1574.8 | -1574.8 | -1574.8 | -1574.8 | 0 | 0 |
| *IRAK4* | -1971.3 | -1966.4 | -1971.3 | -1966.4 | 9.8* | 9.8* |
| *TRAF3* | -2492.8 | -2490.4 | -2493.1 | -2490.4 | 4.8 | 5.4 |
| *RIPK1* | -3067.3 | -3067.3 | -3067.2 | -3067.2 | 0 | 0 |
| *TAB1* | -2323.6 | -2323.6 | -2323.6 | -2323.6 | 0 | 0 |
| *TAB2* | -2818.0 | -2818.0 | -2818.0 | -2818.0 | 0 | 0 |
| *IKKα* | -3225.9 | -3225.9 | -3225.9 | -3225.9 | 0 | 0 |
| *IKKβ* | -2710.1 | -2710.1 | -2710.1 | -2710.1 | 0 | 0 |
| *MKK6* | -1427.5 | -1427.5 | -1427.5 | -1427.5 | 0 | 0 |
| *MKK4* | -1630.0 | -1630.0 | -1630.0 | -1630.0 | 0 | 0 |
| *MKK7* | -2321.4 | -2320.2 | -2321.7 | -2320.2 | 2.4 | 3 |
| *MEK1* | -490.6 | -490.6 | -490.6 | -490.6 | 0 | 0 |
| *MAPK1* | -1370.1 | -1370.1 | -1370.1 | -1370.1 | 0 | 0 |
| *MAPK9* | -1745.5 | -1745.5 | -1745.5 | -1745.5 | 0 | 0 |
| *MAPK10* | -2000.2 | -1998.9 | -2000.6 | -1998.9 | 2.6 | 3.4 |
| *MAPK14* | -1518.4 | -1518.4 | -1518.4 | -1518.4 | 0 | 0 |
| *IRF3* | -1915.3 | -1913.8 | -1915.4 | -1913.8 | 3 | 3.2 |
| *IRF5* | -2238.1 | -2237.4 | -2238.1 | -2237.4 | 1.4 | 1.4 |
| *IRF7* | -2205.9 | -2205.9 | -2205.9 | -2205.9 | 0 | 0 |
| *FOS* | -1649.3 | -1649.3 | -1649.3 | -1649.3 | 0 | 0 |
| *JUN* | -1305.2 | -1305.2 | -1305.2 | -1305.2 | 0 | 0 |

*p<0.05, †statistical significance at FDR test of q=0.05
