# Supplementary material for: Evolutionary patterns of Toll-like receptor signaling pathway genes in the Suidae
Source: BMC Evol Biol. 2016 Feb 9;16:33. doi: 10.1186/s12862-016-0602-7 (PMC4748524; doi:10.1186/s12862-016-0602-7)
Supplement: Additional file 6: Table S5. — Bivariate correlations among variables. (DOCX 13 kb) [file 12862_2016_602_MOESM6_ESM.docx]

**Table S5.** Bivariate correlations among variables

|  |  | **ω** | ***dn*** | ***ds*** | **Position** | **PPI** | **ENC** | **PLENGTH** | **L3’UTR** |
| --- | --- | --- | --- | --- | --- | --- | --- | --- | --- |
| **ω** | *ρ* |  | 0.868 | -0.054 | -0.625 | -0.624 | 0.219 | 0.599 | -0.162 |
|  | *P*_raw |  | <.0001 | 0.7650 | <.0001 | 0.0001 | 0.2216 | 0.0002 | 0.3685 |
|  | *P*_FDR |  | 0.0005 | 0.8380 | 0.0005 | 0.0005 | 0.3266 | 0.0007 | 0.4690 |
| ***dn*** | *ρ* |  |  | 0.364 | -0.611 | -0.674 | 0.035 | 0.672 | -0.259 |
|  | *P*_raw |  |  | 0.0374 | 0.0002 | <.0001 | 0.8446 | <.0001 | 0.1452 |
|  | *P*_FDR |  |  | 0.0806 | 0.0007 | 0.0005 | 0.8759 | 0.0005 | 0.2710 |
| ***ds*** | *ρ* |  |  |  | -0.099 | -0.245 | -0.465 | 0.269 | -0.250 |
|  | *P*_raw |  |  |  | 0.5846 | 0.1692 | 0.0064 | 0.1303 | 0.1609 |
|  | *P*_FDR |  |  |  | 0.7117 | 0.2787 | 0.0163 | 0.2606 | 0.2787 |
| **Position** | *ρ* |  |  |  |  | 0.552 | -0.393 | -0.654 | 0.193 |
|  | *P*_raw |  |  |  |  | 0.0009 | 0.0237 | <.0001 | 0.2812 |
|  | *P*_FDR |  |  |  |  | 0.0028 | 0.0553 | 0.0005 | 0.3756 |
| **PPI** | *ρ* |  |  |  |  |  | -0.083 | -0.529 | 0.193 |
|  | *P*_raw |  |  |  |  |  | 0.6460 | 0.0051 | 0.2817 |
|  | *P*_FDR |  |  |  |  |  | 0.7537 | 0.0042 | 0.3756 |
| **ENC** | *ρ* |  |  |  |  |  |  | 0.226 | -0.017 |
|  | *P*_raw |  |  |  |  |  |  | 0.2064 | 0.9250 |
|  | *P*_FDR |  |  |  |  |  |  | 0.3211 | 0.9250 |
| **PLENGTH** | *ρ* |  |  |  |  |  |  |  | -0.051 |
|  | *p*_raw |  |  |  |  |  |  |  | 0.7781 |
|  | *p*_FDR |  |  |  |  |  |  |  | 0.8380 |

*ρ* is correlation coefficient

*P*_raw is p value before FDR correction

*P*_FDR is p value after FDR correction
